# DNA repair in cancer initiation, progression, and therapy—a double-edged sword

**DOI:** 10.1007/s13353-019-00516-9

**Published:** 2019-08-30

**Authors:** Katarzyna Kiwerska, Krzysztof Szyfter

**Affiliations:** Institute of Human Genetics, Polish Academy of Sciences, Strzeszynska 32, 60-479 Poznan, Poland

**Keywords:** DNA repair, Polymorphism of DNA repair genes, Carcinogenesis, Resistance to chemo- and radiotherapy

## Abstract

Genomic and mitochondrial DNA molecules are exposed continuously for a damaging activity of chemical, physical, and internal genotoxicants. When DNA repair machinery is not working efficiently, the generation of DNA lesions and mutations leads to carcinogenic transformation. The high number of mutation going up to 10^5^ per cell was recognized as a driving force of oncogenesis. Moreover, a high activity of DNA repair genes was hypothesized as a predisposition to metastasis. DNA repair potential has to be taken into account attempting to chemo- and/or radiotherapy. A low activity of DNA repair genes makes tumor cells more sensitive to therapy, but on the other hand, non-tumor cells getting lesions could form second primary cancer. Contrary, high activity of DNA repair genes counteracts attempted therapy. It means an individualized therapy based on recognition of DNA repair potential is recommended.

## DNA repair pathways

Genomic and mitochondrial DNA molecules are exposed continuously for the damaging activity of numerous exogenous and internal genotoxicants. Hence, organisms established a defense system known as the DNA repair process (broader term: DNA damage response abbreviated as DDR). The importance of this field was recognized by the Nobel Committee who awarded Thomas Lindahl (UK), Paul Modrich (USA), and Aziz Sancar (USA) in 2015. The Nobel Prize was given jointly “for mechanistic studies of DNA repair.”

**Fig. 1:**
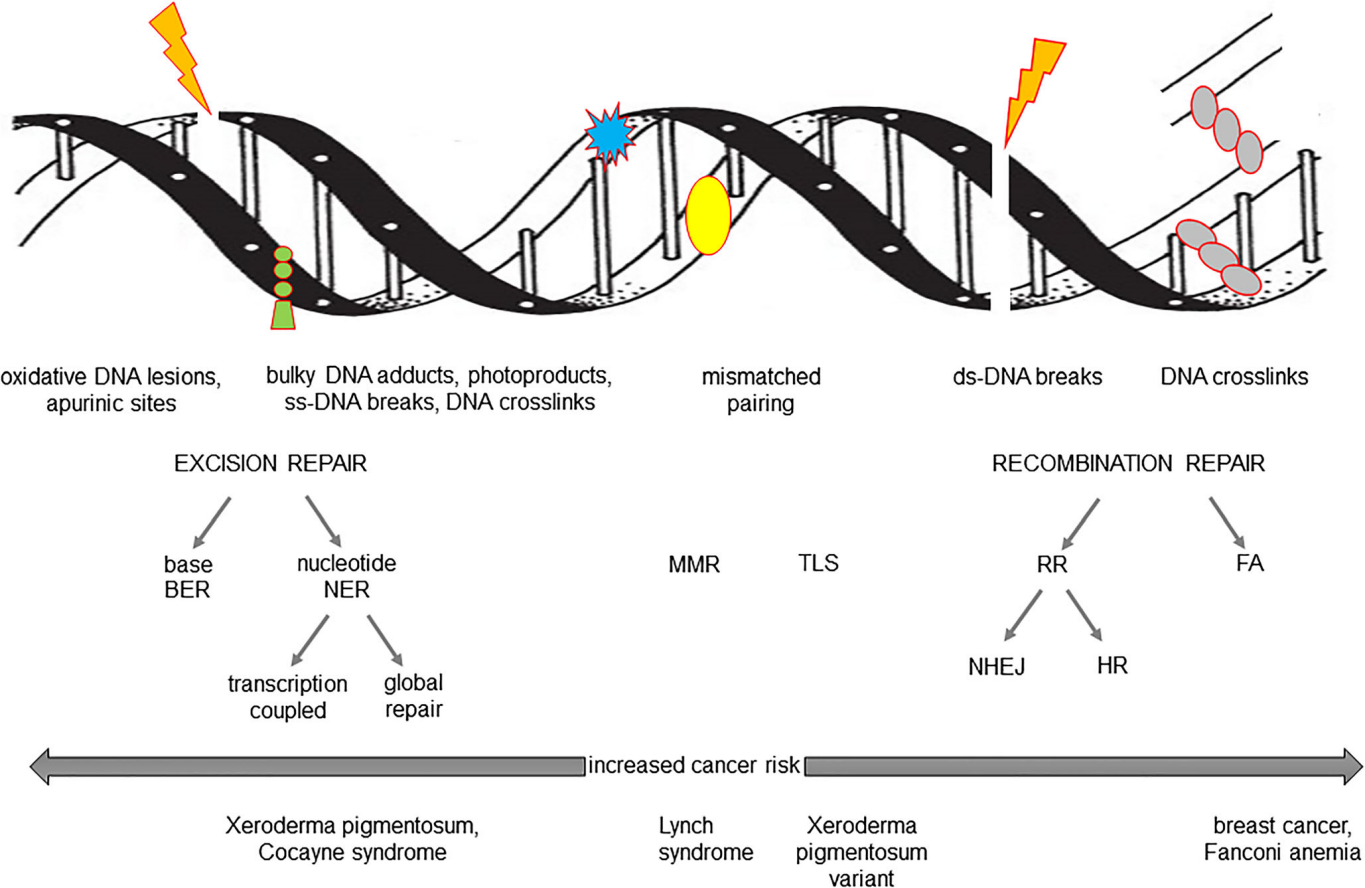
DNA repair pathways. *MMR*, mismatch repair; *TLS*, translesion DNA repair; *RR*, recombination repair; *NHEJ*, non-homologous end joining; *HR*, homologous recombination; *FA*, Fanconi anemia

Protecting the human genome against damage (DNA lesions, mutations, DNA strand breaks, interstrand, and DNA-protein links) provides genome stability and indirectly chromosome maintenance. DNA repair process operates through parallel pathways adjusted to the type of damage and cell cycle. Excision repair (BER, base excision repair; and NER, nucleotide excision repair), mismatch repair (MMR) and recombination repair (NHEJ, non-homologous end joining; and HR, homologous recombination) could be recognized as major pathways operating in all living cells. Schematic presentation of DNA repair pathways is shown in Fig. 1 (Sarasin and Kauffmann 2008; Tian et al. 2015). At this point, it has to be admitted that enzymes involved in particular DNA repair pathways could be reciprocally replaceable. This means that the categorization of DNA repair pathways is not absolute (Shafirovich and Geacintov 2017).

Heterogeneity of DNA repair was established in the last decades of the 20th century. First, it was found that such DNA lesions as apurinic sites, single-strand DNA breaks (SSB), and little base distortions are repaired faster than some photoproducts and double-strand DNA breaks (DSB). Later on, Hanawalt and his team have discovered preferential repair of active DNA segments as compared with global DNA repair (Hanawalt 2012). Unrepaired DNA damage may direct the cell to apoptosis pathway but also, if not successful, may lead to mutation and initiation of carcinogenesis.

An individual DNA repair efficacy varies in the human population that is a consequence of genetic polymorphisms occurring also in DNA repair genes. Polymorphic gene variants are responsible for a moderate variability of DNA repair potential. A strong deficiency of DNA repair is causative for initiating diseases such as Xeroderma pigmentosum, Ataxia telangiectasia, Bloom syndrome, Cockayne Syndrome, trichothiodystrophy, and Nijmegen breakage syndrome. A high incidence of certain types of cancer is attributed to the mentioned diseases (e.g., skin cancer in Xeroderma pigmentosum, Dupuy and Sarasin 2015). Consequently, the genes linked to the mentioned diseases are recognized as “high penetration genes.”

DNA repair variability found in the last decades of the twentieth century was then associated with sensitivity to mutagens and carcinogens, and an individual susceptibility to develop cancer (Alberg et al. 2013). Further, a low DNA repair potential was attributed to an increased cancer risk and the genes staying behind it are known as “low penetration genes.” This attribution recognized as one of the factors of cancer genetic risk was demonstrated for several carcinogens and various cancer types. A good example is a frequent, low DNA repair potential among lung or laryngeal cancer developed in tobacco smokers exposed to tobacco smoke carcinogens (Danoy et al. 2008). Nevertheless, within polymorphisms, gene variants known as “risk genes” or “at risk genes,” determining low DNA repair potential, were found. On the other hand, gene variants associated with DNA repair potential were denominated as “protective genes.” It should be admitted that a number of publications dealing with genetic polymorphism of DNA repair genes and cancer risk have brought a plethora of data, occasionally conflicting. Only recently the published meta-analyses have shown that individual genes do not increase considerable cancer risk but coexistence of some gene variants does (Li et al. 2014; Chen et al. 2014). Altogether, a strong link between low DNA repair potential and increased risk to develop cancer seems to be well established and it will be not discussed further in this review.

## DNA repair in cancer initiation and progression

Initially, an interest in the role of DNA repair in cancer was limited, assuming that its substantial role is linked to coping with DNA damage removal. Now, it is perfectly known that the lesions not eliminated are accumulating, driving a cell to carcinogenic transformation. An increasing number of molecular malformations gave rise to postulate an occurrence of the mutator phenotype (Loeb et al. 2008). An estimation goes up to 10^5^ mutations per cancer genome. As not all somatic mutations contribute equally to carcinogenesis, they were divided into “driver” and “passenger” mutations. The used term explains their significance in an obvious way (Stratton et al. 2009). Mutations in DNA repair genes are not frequently listed among cancer driver mutations. Deregulation of DNA repair in cancer cells should be considered rather as an alteration of DNA damage response, which includes mutations of DNA repair, cell cycle, and apoptosis genes (Knijnenburg et al. 2018). In any case, transformed cells remain under unbroken supervision of DNA repair machinery, protecting against the terminal accumulation of damage directing the cell to death (Roos and Kaina 2013). Therefore, a changed DNA repair potential should rather be estimated in relation to genetic polymorphism and not to gene mutations.

As expected, some papers reported that the same DNA repair deficiency that once initiated carcinogenesis is detectable throughout cancer progression. A reduced DNA repair function can be inherited or induced by an exposure to genotoxicants. Though, it seems cancer patients with low DNA repair function are likely to have a poorer prognosis.

On the other hand, it was shown that a low activity of the DNA repair system in tumor tissue seems to be a better prognostic factor for longer survival. To give a supporting example, Vodička’s group was studying base excision DNA repair induced by 5-fluorouracil treatment of colon cancer patients. The metabolized 5-fluorouracil incorporates uracil into the DNA molecule that is later removed by the BER DNA repair mechanism. The removal process was determined in 123 paired samples, derived from colon tumor cells and from adjacent non-tumor material. Having establishing interindividual differences, the authors concluded that BER DNA repair appears to be a major driving force in malignant transformation and further can be applied as a useful prognostic biomarker. Better applicability is attributed to the BER parameter determined in non-tumor adjacent material. Nevertheless, the overall survival of the studied patients was even better in the presence of a decreased BER DNA repair mechanism (Vodenkova et al. 2018).

The impact of polymorphic DNA repair genes participating in excision repair was studied in various cancer types. A large study by Smolarz et al. (2019), analyzing the distribution of *XRCC1*, *XRCC2*, *XPD*, and *RAD5*1 gene variants in 300 breast cancer patients and 300 controls, did not observe an association between variants determining poor DNA repair potential with cancer progression. However, one of the mentioned genes, namely *XRCC1*, was taken under study in the same cancer (*n* = 118) but with respect to the other polymorphic site. It was established that the gene variant *XRCC1* (C194T) can inhibit proliferation and invasion, and is promoting apoptosis. Moreover, a higher frequency of *XRCC1* (C194T) is linked with lymphatic metastasis. Hence, the alternative polymorphic site at *XRCC1* is playing a double role in breast cancer with antitumor activity and promotion of metastasis as well (Li et al. 2018). Next, another study done on almost the same set of genes but on myeloproliferative neoplasm (*n* = 133) treated with hydroxyurea has established a role of BER genes in progression and clinical evolution of neoplasm (Azevedo et al. 2018). Further, the study by Santana et al. (2016) has shown overexpression of *AP-1* and *XRCC-1* in oral tongue squamous cell carcinoma (*n* = 82). A high expression of *XRCC-1* was significantly associated with early tumor stage. The study also linked the overexpression of APE-1 to cancer aggressiveness. Altogether, the studies on BER genes in cancer progression provided rather conflicting results.

BER DNA repair deficiency is not necessarily connected with genetic polymorphism. Other possible mechanisms of deficiency include epigenetic downregulation, gene expression regulation by microRNA, and interference with cell cycle regulating elements, including *TP53* pathway (Kaina et al. 2018). The latter question was a matter of the studies of the G. Dianov laboratory. Supposing that unrepaired DNA SSB can be converted into DSB that challenges genome and chromosome stability, it was shown that accumulation of SSB downregulates protein APE1 responsible for DNA incision during BER. At the same time, an impairment of *TP53* common in cancer can compensate for APE1 deficiency and stimulate the processing of SSB by BER mechanism (Poletto et al. 2016).

Similar studies were performed concerning genes involved in nucleotide excision DNA repair. Seivert et al. (2014) studied the expression of DNA repair genes in advanced head and neck cancer. One of the findings was that *XPF* forming a heterodimer with *ERCC1* exhibits a large range of expression. A low expression indicates a better response to chemoradiotherapy and vice versa. Peng et al. (2018) attempted to determine the role of *ERCC1* as another NER participating gene in esophageal squamous cell carcinoma (*n* = 103). Inhibition of apoptosis by overexpressed *ERCC1* was shown and explained as unfavorable prognostic factor. The role of polymorphic variants of eight NER genes was studied also in non-small cell lung cancer. Two of them, namely *ERCC1* rs12924 AG/GG and *XPC* rs2229090 GC/CC, have been proven to predict disease progression free survival and overall survival. The effect was better pronounced in adenocarcinoma than in squamous cell carcinoma (Zhang et al. 2017). Further, Jacobsen et al. (2017) studying the expression of *ERCC1* in prostate cancer established an association of gene overexpression with the formation of chromosome aberrations. Some gene fusions and deletions of *PTEN*, 6q, 5q, and 3p have been shown as significant players in cancer progression. Genomic instability was mostly driven by low-grade tumors and indicated a poor prognosis.

A significance of the mismatch repair mechanism in cancer progression did not attract too much research attention. However, MMR is a critical mechanism involved in maintaining microsatellite stability that in turn is associated with chromatin organization and recombination. Microsatellite mutational variability could be followed by inter- and intra-tumor heterogeneity. Microsatellite variability seems to be applicable to differentiate between hereditary and carcinogen-dependent colorectal cancer (Shah et al. 2010). A reduced expression of MMR genes *MSH6/MSH2* estimated on mRNA and protein level was established to promote pituitary tumor growth (Uraki et al. 2018). The latter finding is consistent with that established by Germano et al. (2018) who have shown that tumors carrying defects in MMR accumulate mutations followed by rapid tumor progression. Nejda et al. (2009) studying three polymorphisms in MMR gene *MLH1* have established a double role of the variant associated increasing a risk to develop nonpolyposis colorectal cancer. Later on, the same gene was found to be associated with a better clinical outcome. The authors did not provide an explanation of this phenomenon.

DSB removal is being studied rather in relation to radio- and chemotherapy, capable to generate double-strand breaks. Recombination is maintaining genomic integrity throughout the cell cycle except for the mitosis phase. Further, DSBs seem to be toxic to the cells that result in chromosome missegregation and the formation of chromosome aberrations (Terasawa et al. 2014).

At this point, it is worth to cite the hypothesis of Alain Sarasin, associating an overexpression of DNA repair genes with metastasis. According to this hypothesis, DNA repair genes signature discriminates between primary and metastasizing tumors. In fact, the difference emerges at the stage of primary tumors where these with high metastatic potential differ from the others mainly by the overexpression of MMR genes (Sarasin and Kauffmann 2008). Then, the overexpressed genes involved in double-strand break repair and surveillance of DNA replication forks provide a room for entering metastasis. The hypothesis of Sarasin was positively verified by Chakraborty et al. (2018). Using The Cancer Genome Atlas, the authors analyzed the expression of genes involved in the DNA mismatch repair. The increased number of gene copies of *MSH2* and *MSH6* affects DNA replication forks and in turn contributes to genome instability, which promotes cancer progression.

## DNA repair in cancer chemo- and radiotherapy

The main target of cancer therapy is the elimination of tumor cells by their surgical removal (not discussed in this review) or by killing tumor cells by means of radio- or chemotherapy. Immunotherapy and targeted therapy are not so common because of still ongoing research and high costs. Limited success in cancer therapy could be linked to the side effects and resistance to radiotherapy. Concerning DNA repair, its efficiency modulates considerable removal of lesions induced by therapy that opposes the expected damage of tumor cells (Toulany 2019).

Recently, a research interest has been focused on the significance of DNA repair in cancer progression and therapy. Cancer therapy employs chemo- and radiotherapy to eliminate or at least to inhibit the growth of tumor cells. One of the ways to get the target is the induction of DNA damage in tumor cells. As chemotherapeutic agents are not sufficiently selective, DNA lesions could emerge in non-tumor cells. Unfortunately, DNA repair pathways are capable to remove lesions also from tumor cells already purposely generated in there. In case of an intensive repair of lesions in tumor cells, they are becoming chemoresistant (Sakthivel and Hariharan 2017). Chemoresistance can be developed against many (all?) cytotoxic drugs such as cisplatin, carboplatin, 5-fluorouracil (5-FU), metotrexate, bleomycin, or docetaxel. Principally, the resistance to chemotherapy is developed to such antimetabolite drugs such as 5-FU and metotrexate. 5-FU is antimetabolite chemotherapeutic acting as a suicide inhibitor of thymidylate synthase converting dUMP to dTMP. Concerning cisplatin, its DNA damaging activity is connected with the formation of DNA adducts that can be converted into inter- and intrastrand cross-links. Hence, platinum chemoresistance is associated with the following DNA repair pathways: nucleotide excision repair, recombination repair, and mismatch repair. Going into details, a survival benefit was established in lung cancer patients with a low level of *ERCC1* involved in excision repair (Martin et al. 2008). Nogueira et al. (2018) studying a response to cisplatin in head and neck squamous cell carcinoma patients have estimated the role of DNA mismatch repair genes polymorphisms in toxicity and response to the drug. An increased risk of therapy failure was established for carriers of some variants of *MSH3* (one genotype), *EXO1* (two genotypes); a partial response only to therapy was observed instead of the expected complete remission.

Studies on genetic polymorphism in genes involved in different mechanisms of DNA repair became very productive in discovering potential DNA repair deficit followed by therapy failure. The statement is applicable both to classical genotyping (Alberg et al. 2013; Smolarz et al. 2019) and to modern biotechnology and bioinformatics (Knijnenburg et al. 2018) Further, this is a way for individualizing therapeutic attempts. Literature survey indicates that variability of response to chemotherapy could be expected in any cancer type.

Cancer treatment with radiotherapy concerning effectiveness also falls under similar rules as chemotherapy. A well-digestive review paper by Hosoya and Miyagawa (2014) suggests to start therapy planning from taking into account abnormalities in the DNA damage response machinery in the cancer cell, supposing DNA repair proceeds normally in non-cancer cells. To give an example, an impact of genetic polymorphism on the efficiency of radiotherapy was found in nasopharyngeal carcinoma for genes involved in base excision repair (Wang et al. 2017). In head and neck cancer, there is a common infection by human papillomavirus (HPV). It was established that cell lines derived from HPV(+) HNSCC are more sensitive for radiation than HPV(−) lines mainly because of a reduced potential to repair double-strand breaks. Though, the standard radiotherapy protocol exposes HPV-infected patients for an increased risk to develop side effects (Nickson et al. 2017).

Radiotherapy-induced DNA damage can lead to apoptosis, inhibition of replication, and generation of second primary tumors. First of all, it is connected with leaving unrepaired double-strand DNA breaks. The measurement of unrepaired DSB could be used to identify patients at greater risk to develop the adverse effects of treatment (Noda 2018).

## Attempts to avoid side effects of chemo- and radiotherapy

Two situations implicate studies on therapy planning to avoid adverse effects of radio- and chemotherapy. The first is that cancer cells already having disrupted DNA repair are compensating it by activating alternative repair pathways (Tian et al. 2015). The second refers to genetic polymorphism responsible for an individual high DNA repair potential (Nogueira et al. 2018). In radiotherapy, such technical solutions as fractionation of radiation dose or better protection of irradiated region were established to eliminate adverse effects (Milecki and Szyfter 2003). Beside it, a few molecular and cellular strategies were developed to change DNA repair potential. Inhibition of an overexpression of DNA repair genes allows for decreasing of adverse effects of DNA repair in tumor cells and further to reduce a drug dose applied to cure cancer (Hosoya N and Miyagawa K 2014). A few proposals managed in terms of molecular biology will be mentioned below.

PARP1 [poly(ADP-ribosyl) polymerase] is a multifunctional enzyme active in DNA repair, maintaining genomic stability and transcription regulation. Because of a strong link of DNA repair with replication, PARP1 inhibitors such as olaparib acting as a competitive inhibitor of PARP enzymes at the catalytic site of PARP1 can increase radiosensitivity. The latter allows to decrease a dose and to avoid undesired effects. Another example of indirect regulation of DNA repair is an application of afatinib as a blocker of epidermal growth factor receptor. As a result, an inhibition of EGFR followed by a higher anticancer activity of cisplatin in HNSCC was observed (Longton et al. 2018). Likely, several inhibitors of all DNA repair pathways applicable to various cancer types acting directly or indirectly on DNA repair genes were established (Hosoya and Miyagawa 2014).

It is worth to turn attention separately on the role of *TP53* gene frequently mutated in many cancers. The loss of function of tumor suppressor gene *TP53* is associated with increased tumor aggressiveness. Restoration of wild-type *TP53* function was demonstrated using gene therapy, antibodies, and small synthetic molecules. Reactivation of *TP53* in tumor cells leads to the increase of radiation susceptibility and helps to improve treatment effectiveness (Bossi and Saccho 2007).

## Conclusions

The idea of the review was to present the significance of the DNA repair process in all stages of cancer development. On one hand, its low potential promotes entering tumorigenesis pathway because of poor efficiency in removing of carcinogen-induced DNA lesions. Further, at the stage of cancer progression, pro- and anti-carcinogenic activities of DNA repair were postulated. Concerning radio- and chemotherapy, a double-edge effect of DNA repair emerges clearly. Hence, dependently of the disease stage the same process of DNA repair is increasing risk or lowering survival. Fortunately, an intensive research provided tools to regulate the risk at all stages (Kaina et al. 2018; Tian et al. 2015).
